# Anti-integrin αvβ6 antibody as a biomarker for diagnosing ulcerative colitis: a nationwide multicenter validation study

**DOI:** 10.1007/s00535-024-02176-x

**Published:** 2024-11-28

**Authors:** Makoto Okabe, Shuji Yamamoto, Masahiro Shiokawa, Tadakazu Hisamatsu, Hajime Yamazaki, Risa Nakanishi, Kensuke Hamada, Hiroki Kitamoto, Takeshi Kuwada, Norimitsu Uza, Aki Sakatani, Toshimitsu Fujii, Masashi Ohno, Minoru Matsuura, Tomoyoshi Shibuya, Naoki Ohmiya, Makoto Ooi, Namiko Hoshi, Kei Moriya, Kiichiro Tsuchiya, Yoshiharu Yamaguchi, Reiko Kunisaki, Masahiro Takahara, Tomohisa Takagi, Tetsuo Takehara, Fumihito Hirai, Kazuki Kakimoto, Motohiro Esaki, Hiroshi Nakase, Fukunori Kinjo, Takehiro Torisu, Shuji Kanmura, Kazuyuki Narimatsu, Katsuyoshi Matsuoka, Hiroto Hiraga, Kaoru Yokoyama, Yusuke Honzawa, Makoto Naganuma, Masayuki Saruta, Yuzo Kodama, Tsutomu Chiba, Hiroshi Seno

**Affiliations:** 1 Department of Gastroenterology and Hepatology, Graduate School of Medicine, Kyoto University, 54 Shogoin Kawahara-cho, Sakyo-ku, Kyoto 606-8507 Japan; 2 Department of Gastroenterology and Hepatology, Kyorin University School of Medicine, Mitaka-shi, Tokyo 181-8611 Japan; 3 Section of Clinical Epidemiology, Department of Community Medicine, Graduate School of Medicine, Kyoto University, Kyoto, 606-8507 Japan; 4 Division of Gastroenterology, Department of Internal Medicine, Asahikawa Medical University, Asahikawa, 078-8510 Japan; 5 Department of Gastroenterology and Hepatology, Tokyo Medical and Dental University, Tokyo, 113-8519 Japan; 6 Department of Medicine, Shiga University of Medical Science, Otsu, 520-2192 Japan; 7 Department of Gastroenterology, Juntendo University School of Medicine, Tokyo, 113-8421 Japan; 8 Department of Advanced Endoscopy, Fujita Health University School of Medicine, Toyoake, 470-1192 Japan; 9 Division of Gastroenterology, Department of Internal Medicine, Graduate School of Medicine, Kobe University, Kobe, 650-0017 Japan; 10 Department of Gastroenterology, Nara Prefecture General Medical Center, Nara, 630-8581 Japan; 11 Department of Gastroenterology, Institute of Medicine, University of Tsukuba, Tsukuba, 305-8576 Japan; 12 Division of Gastroenterology, Department of Internal Medicine, Aichi Medical University School of Medicine, Nagakute, 480-1195 Japan; 13 Inflammatory Bowel Disease Center, Yokohama City University Medical Center, Yokohama, 232-0024 Japan; 14 Department of Gastroenterology and Hepatology, Dentistry and Pharmaceutical Sciences, Okayama University Graduate School of Medicine, Okayama, 700-8558 Japan; 15 Department of Molecular Gastroenterology and Hepatology, Kyoto Prefectural University of Medicine, Kyoto, 602-8566 Japan; 16 Department of Gastroenterology and Hepatology, Osaka University Graduate School of Medicine, Suita, Osaka 565-0871 Japan; 17 Department of Gastroenterology, Faculty of Medicine, Fukuoka University, Fukuoka, 814-0180 Japan; 18 Second Department of Internal Medicine, Osaka Medical and Pharmaceutical University, Takatsuki, 569-8686 Japan; 19 Division of Gastroenterology, Department of Internal Medicine, Faculty of Medicine, Saga University, Saga, 849-8501 Japan; 20 Department of Gastroenterology and Hepatology, Sapporo Medical University School of Medicine, Sapporo, 060-8543 Japan; 21 Center for Gastroenterology, Urasoe General Hospital, Urasoe, 901-2102 Japan; 22 Department of Medicine and Clinical Science, Graduate School of Medical Sciences, Kyushu University, Fukuoka, 812-8582 Japan; 23 Division of Digestive and Lifestyle Diseases, Kagoshima University Graduate School of Medical and Dental Sciences, Kagoshima, 890-8520 Japan; 24 Department of Internal Medicine, National Defence Medical College, Tokorozawa, 359-8513 Japan; 25 Division of Gastroenterology and Hepatology, Department of Internal Medicine, Toho University Sakura Medical Center, Sakura, 285-8741 Japan; 26 Department of Gastroenterology and Hematology, Hirosaki University Graduate School of Medicine, Hirosaki, 036-8563 Japan; 27 Department of Gastroenterology, Kitasato University School of Medicine, Kanagawa, 252-0375 Japan; 28 Division of Gastroenterology and Hepatology, Third Department of Internal Medicine, Kansai Medical University, Osaka, 573-1010 Japan; 29 Division of Internal Medicine, Department of Gastroenterology and Hepatology, The Jikei University School of Medicine, Tokyo, 105-8471 Japan; 30 Department of Gastroenterology and Hepatology, Kansai Electric Power Hospital, Fukushima-ku, Osaka, 553-0003 Japan

**Keywords:** Anti-integrin αvβ6 antibody, Ulcerative colitis, Colonic Crohn’s disease, Diagnostic biomarker

## Abstract

**Background:**

A serum biomarker for diagnosing ulcerative colitis (UC) remains to be established. Although we recently reported an anti-integrin αvβ6 antibody (V6 Ab) for diagnosing UC with high sensitivity and specificity, no large-scale validation study exists. This study aimed to validate the diagnostic value of V6 Ab for UC using a nationwide multicenter cohort study.

**Methods:**

We measured V6 Ab titers in patients definitively diagnosed with UC, Crohn’s disease (CD), or other gastrointestinal disorders (OGDs). The primary outcome was the diagnostic value of V6 Ab. Secondary outcomes were factors associated with false-negative results in patients with UC and false-positive results in patients without UC and the heterogeneity of the diagnostic value of V6 Ab among the participating facilities.

**Results:**

We enrolled 1241, 796, and 206 patients with UC, CD, and OGD, respectively, from 28 Japanese high-volume referral centers. The diagnostic sensitivity of V6 Ab for UC was 87.7%, and its specificities for CD and OGDs were 82.0% and 87.4%, respectively. Multivariable logistic regression analysis showed that false-negative results were associated with older age at the time of sample collection, current smokers, lower partial Mayo score, and not receiving advanced therapies in patients with UC, and false-positive results were associated with colonic CD in patients with CD. No factor was associated with false-positive results in patients with OGDs. There were no significant differences in the diagnostic value of V6 Ab among the centers.

**Conclusions:**

The diagnostic value of V6 Ab for UC was validated in the large-scale nationwide multicenter study.

**Supplementary Information:**

The online version contains supplementary material available at 10.1007/s00535-024-02176-x.

## Introduction

Inflammatory bowel disease (IBD) is an idiopathic, chronic, and inflammatory disorder mainly affecting the gastrointestinal tract and is classified into two subtypes: ulcerative colitis (UC) and Crohn’s disease (CD) [1]. UC is characterized by a relapsing and remitting mucosal inflammation, starting in the rectum and extending to the proximal segments of the colon [2]. Approximately 0.1–0.5% of the population in highly developed countries reportedly have UC [3]. Additionally, UC incidence has been increasing in newly industrialized countries where its prevalence is lower than that in Western countries [3]. Therefore, UC is a global burden [4].

UC is characterized by symptoms, such as diarrhea, rectal bleeding, abdominal pain, fever, and loss of body weight [1, 2], which are often indistinguishable from the symptoms of other gastrointestinal diseases, including CD, gastrointestinal lesions due to systemic inflammatory disease, infectious enterocolitis, and drug-induced gastroenteropathy. There is no reference standard for diagnosing UC; hence, UC is diagnosed based on a combination of clinical, biochemical, stool, endoscopic, and histological investigations [5].

Integrin αvβ6 is a heterodimer consisting of αv and β6 integrin subunits and is expressed on the colonic epithelial cell surface of patients with UC [6]. Integrin αvβ6 binds to the extracellular matrix [7] and is reported to play an important role in maintaining epithelial barrier functions [8]. We recently reported anti-αvβ6 integrin antibodies (V6 Abs) with high sensitivity and specificity for diagnosing UC in Japanese adult [6] and pediatric [9] patients, which were further confirmed in Swedish [8], North American [10, 11], and Italian [12] cohorts. However, the numbers of enrolled patients and participating facilities were relatively small in these studies, and the control group consisted of healthy persons without gastrointestinal symptoms. Therefore, this study aimed to conduct a large-scale nationwide validation study on the diagnostic value of V6Ab for UC.

## Methods

### Study design and patients

Between January 11, 2022, and May 31, 2023, a nationwide multicenter observational study recruited patients with gastrointestinal diseases at 28 Japanese high-volume referral centers (Table S1).

The inclusion criteria were (1) patients aged ≥ 18 years at the time of sample collection, (2) established diagnosis of UC, CD, and other gastrointestinal disorders (OGDs) at each local institution, and (3) obtained informed consent. The exclusion criteria were (1) inflammatory bowel disease unclassified (IBDU; chronic colitis not differentiated between UC and CD), (2) opted-out consent, and (3) patients judged to be ineligible by the investigators.

In all cases, the diagnosis of UC and CD was established according to standardized criteria based on prior clinical assessment and radiological, endoscopic, and histological findings [14]. The diagnostic criteria for each control disease are listed in Table S2.

We collected serum samples from the enrolled patients and measured V6 Ab titers using an anti-integrin αvβ6 enzyme-linked immunosorbent assay (ELISA) kit (Cat. No. 5288, Medical & Biological Laboratories Co., Ltd., Tokyo, Japan) [15] by blinded central assessment. Baseline demographic and clinical variables and medications at the time of sample collection were retrieved from the medical record, including sex; age at the time of sample collection; smoking status; age at disease onset; disease extension in UC; disease location and behavior in CD; laboratory data, including C-reactive protein (CRP) and leucine-rich alpha 2 glycoprotein levels; and medications.

This study was performed according to the Declaration of Helsinki and approved by the Ethics Committee of Kyoto University Graduate School and Faculty of Medicine (protocol number: R3135). Before collecting the serum samples, patients were informed about the measurement of the V6 Ab and provided written consent. Preserved serum samples from another study were allowed if the patient did not opt out of the secondary usage of the samples obtained after diagnosis of the gastrointestinal disease.

### Outcome measures

The primary outcome was the diagnostic value of V6 Ab. Secondary outcomes were factors associated with false-negative results in patients with UC, factors associated with false-positive results in patients without UC, and the diagnostic value of V6 Ab at each participating facility.

### Definitions and assessment

Disease extension (UC) and disease location and behavior (CD) were categorized according to the Montreal classification [16]. Clinical disease activity of UC and CD was measured using the partial Mayo score [17] and the Harvey–Bradshaw index [18], respectively. The endoscopic activity was assessed using the Mayo endoscopic subscore (MES). [17]

### ELISA

As described above, we used an anti-integrin αvβ6 ELISA Kit to measure serum V6 Ab titer [15]. Serum samples were diluted to 1:100 with a reaction buffer. The recombinant monoclonal human V6 Abs were serially diluted from 0.781 to 200.0 U/mL with the reaction buffer as the zero standard. ELISA plates were incubated for 60 min at room temperature with 100 μL of serum diluted 100 times or standard material. After washing, 100 μL of horseradish peroxidase-conjugated antibody against human IgG was added, and the plates were incubated for 60 min at 20 °C. After another washing, 100 μL of 3,3′,5,5′-tetramethylbenzidine was added to each well and incubated at room temperature for 20 min. At 450 nm, absorbance was measured following the addition of 100 μL of stop solution. The antibody concentration of each sample was determined using a calibration curve generated based on the optical density value of the standard material through a four-parameter logistic regression. Samples with optical densities below the lower limit of the calibration curve were considered to have a concentration of 0 U/mL. The cut-off value for the diagnosis of UC was 1.64 U/mL, calculated as the mean + 3 standard deviations (SDs) of 83 serum samples from healthy volunteers as determined by the manufacturer.

### Statistical analysis

Continuous variables were represented as mean ± SD. Categorical variables were represented as median (minimum–maximum). Univariable and multivariable logistic regression analyses were used to evaluate factors associated with false-positive and false-negative results in patients with UC and without UC, respectively. *I*^2^ values were calculated to test for heterogeneity in diagnosing UC using V6 Ab between the participating centers. The chi-squared tests were used to compare the positive rates of V6 Ab among patients with different categories. A *p*-value of < 0.05 was considered statistically significant. STATA version 18.0 (Stata Corporation, TX, USA) and GraphPad Prism version 9.4.1 (GraphPad Software, San Diego, CA, USA) were used for analysis.

## Results

**Fig. 1:**
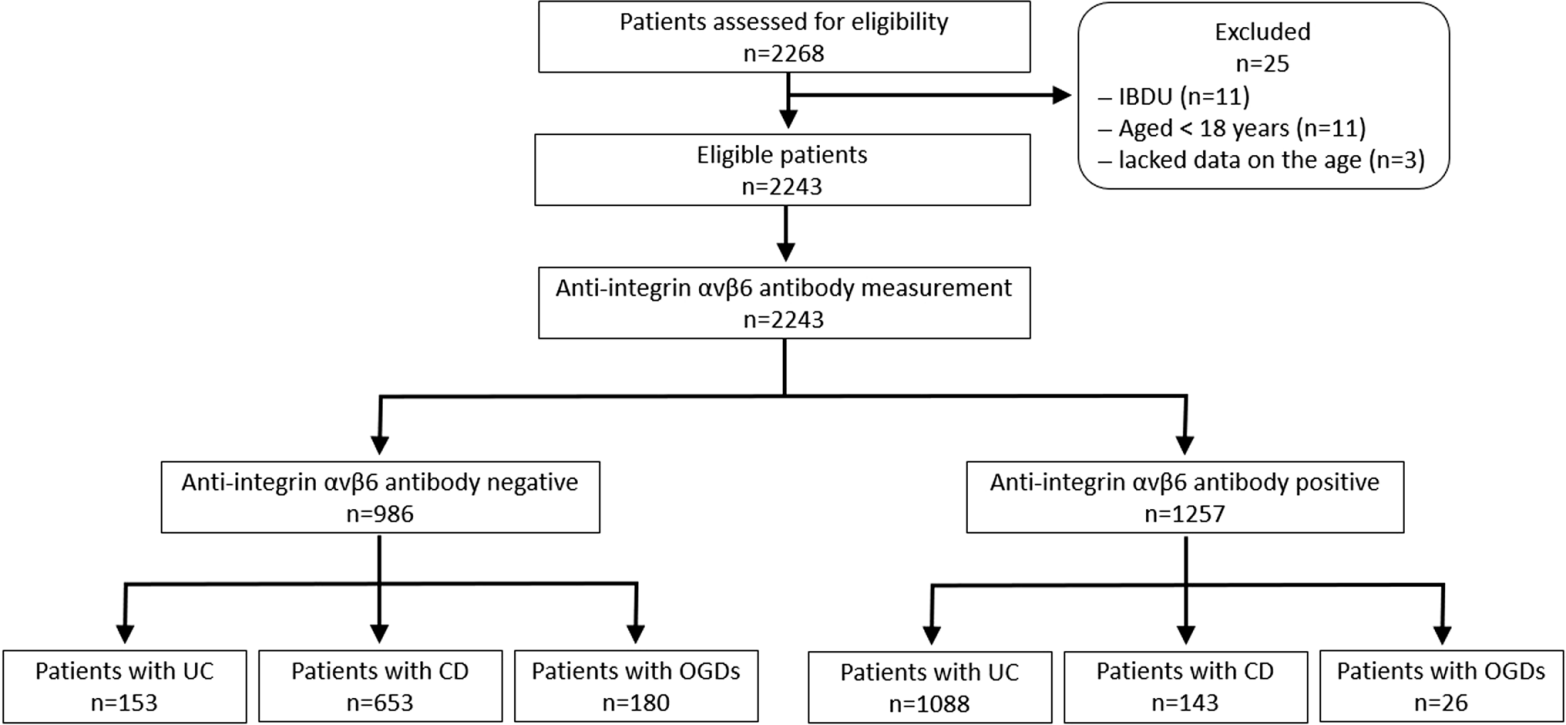
Patient flowchart

Overall, 2268 patients were enrolled, of which 11 patients with IBDU, 11 patients aged < 18 years at the time of sample collection, and 3 patients without data on their age were excluded, resulting in 2243 patients for the analysis (Fig. 1). Of the 2243 analyzed serum samples, 311 were preserved, and 1932 were collected after obtaining informed consent. The disease entities of the enrolled patients are indicated in Table 1. The numbers of patients with UC, CD, and OGDs were 1241, 796, and 206, respectively. OGDs included inflammatory diseases (108 cases), gastrointestinal tumors (30 cases), drug-induced gastrointestinal disorders (20 cases), and others (48 cases).

**Table 1:** Disease entities of the enrolled patients

Disease entity	*n*
**UC**	**1241**
**CD**	**796**
**OGDs**	**206**
*Inflammatory diseases*	**108**
Behcet’s disease	76
Chronic enteropathy associated with *SLCO2A1* gene	3
Cronkhite-Canada syndrome	10
Familial Mediterranean fever	5
Vasculitis (IgA vasculitis and eosinophilic granulomatosis with polyangiitis, Takayasu’s arteritis)	3
Eosinophilic gastroenteropathy	7
Congenital immunodeficiency	4
*Gastrointestinal tumors*	**30**
Colonic adenocarcinomas or adenomas	26
Neuroendocrine tumors	3
Gastrointestinal lymphoma	1
*Drug-induced gastrointestinal disorders*	**20**
Immune-related adverse events (irAE)	17
Olmesartan associated enteropathy	1
Non-steroidal anti-inflammatory drug-induced enteropathy	1
Microscopic colitis	1
*Others*	**48**
Infectious enterocolitis	6
Radiation enterocolitis	2
Ischemic colitis	9
Irritable bowel syndrome	12
Colonic diverticular disease	4
Gastroesophageal reflux disease	3
Non-specific enterocolitis	6
Malabsorption syndrome	1
Amyloidosis	1
Phlebosclerosis	1
Bowel obstruction	1
Small intestinal bleeding	1
Colonic angiectasia	1

*n* number of patients, *UC* ulcerative colitis, *CD* Crohn’s disease, *OGDs* other gastrointestinal diseases

Bold or Italic was made to divide major categories from subcategories

Patients’ baseline characteristics are shown in Table 2. The mean ages at the time of sample collection of patients with UC, CD, and OGDs were 46.5 ± 16.4, 41.6 ± 14.1, and 55.5 ± 17.6 years, respectively. In patients with UC, the number of patients with proctitis (E1), left-sided (E2), pancolitis (E3), and post-colectomy were 128, 264, 754, and 22, respectively; median partial Mayo score was 0 (0–9); median MES was 1(0–3); mean serum CRP level was 2.2 ± 6.1 mg/L, and 107 (13.5%) and 445 (35.8%) patients were treated with steroids and advanced therapies, respectively. The number of patients with CD with ileal (L1), colonic (L2), and ileocolonic (L3) CD were 218, 91, and 431, respectively; the median Harvey–Bradshaw index was 1 (0–18); mean serum CRP level was 3.5 ± 9.2 mg/L; and 48 (6.0%) patients were treated with steroids and 547 (68.7%) with advanced therapy.

**Table 2:** Patients’ baseline characteristics

	UC (*n* = 1241)	CD (*n* = 796)	OGDs (*n* = 206)
**Sex**			
Male	673 (54.2%)	544 (68.3%)	101 (49.0%)
Female	568 (45.8%)	252 (31.7%)	105 (51.0%)
**Age at sample collection **[years, (mean ± SD)]	46.5 ± 16.4	41.6 ± 14.1	55.5 ± 17.6
**Smoking status** (non-smoker/ ex-smoker/ current smoker)	715/ 223/ 90	443/95/76	82/31/15
**Age at onset **[years, (mean ± SD)]	35.0 ± 15.5	28.2 ± 13.0	49.3 ± 20.3
**Disease extension** (E1/ E2/ E3/ post-colectomy)	128/ 264/ 754/ 22	NA	NA
**Disease location** (L1/ L2/ L3)	NA	218/ 91/ 431	NA
**Disease behavior** (B1/ B2/ B3/p)	NA	347/ 290/ 98/270	NA
**Partial Mayo score** [median (range)]	0 (0–9)	NA	NA
**Mayo endoscopic subscore** [median (range)]	1 (0–3)	NA	NA
**Harvey–Bradshaw index** [median (range)]	NA	1 (0–18)	NA
**CRP** [mg/L, (mean ± SD)]	2.2 ± 6.1	3.5 ± 9.2	11.7 ± 27.1
**LRG** [μg/mL, (mean ± SD)]	13.6 ± 6.2	15.5 ± 7.4	18.0 ± 9.5
**Treatment**			
5-aminosalicylic acid or sulfasalazine	977	482	NA
Thiopurine	291	287	NA
Steroids	167	48	NA
Tacrolimus	17	4	NA
Advanced therapy (TNFi/ VDZ/ UST/ RIS/JAKi)	445 (172/ 96/ 82/ 0/ 101)	547 (350/ 24/ 167/ 8/ 1)	NA
Post total colectomy	22	NA	NA
No treatment	28	6	NA

*UC* ulcerative colitis, *CD* Crohn’s disease, *OGDs* other gastrointestinal diseases, *NA* not applicable, *CRP* C-reactive protein, *LRG* leucine-rich alpha 2 glycoprotein, *TNFi* TNF inhibitors, *VDZ* vedolizumab, *UST* ustekinumab, *RIS* risankizumab, *JAKi* JAK inhibitors

**Fig. 2:**
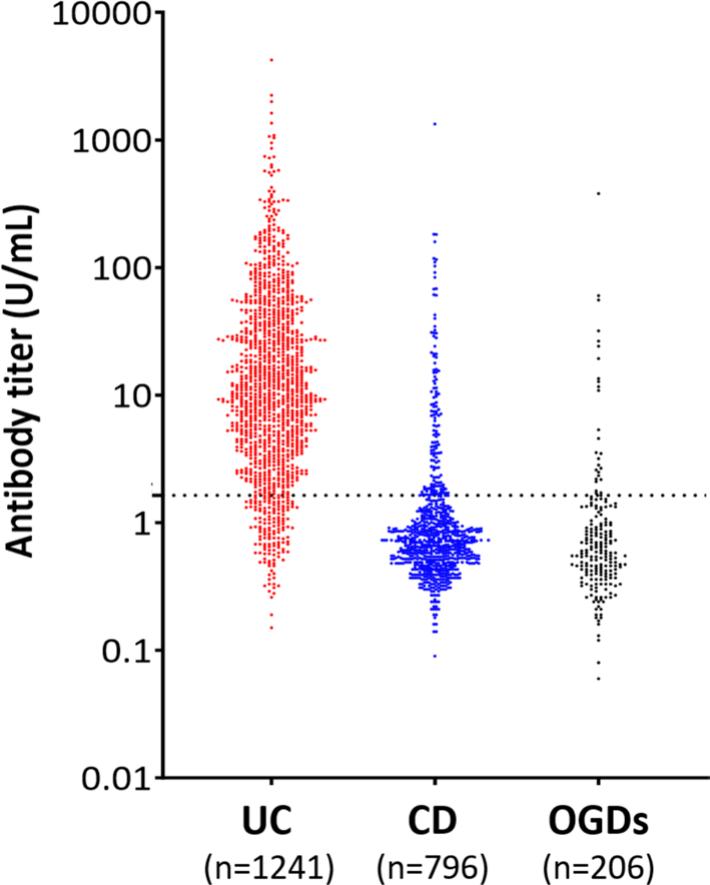
Serum anti-integrin αvβ6 antibody titers in patients with UC, CD, and OGDs. V6 Ab titers were measured using an enzyme-linked immunosorbent assay kit. The cut-off value of 1.64 U/mL, defined as the mean + 3SDs of 83 serum samples from healthy volunteers as determined by the manufacturer, is indicated by a dashed line. *n* total number of patients in each group. *UC* ulcerative colitis, *CD* Crohn’s disease, *OGDs* other gastrointestinal diseases

The positive detection rates of V6 Ab in patients with UC, CD, and OGD were 87.7% (1088/1241), 18.0% (143/796), and 12.6% (26/206), respectively (Fig. 2). The positive detection rates of each disease entity of OGDs are shown in Figure S1. The diagnostic sensitivity of V6 Ab for UC was 87.7% [95% confidence interval (CI): 85.7–89.4], and its specificities were 82.0% (95% CI: 79.2–84.6) for CD and 87.4% (95% CI: 82.1–91.6) for OGDs.

Univariable logistic regression analysis (Table S3) showed that false-negative results of V6 Abs in patients with UC were associated with age at the time of sample collection, current smokers, age at onset, proctitis, partial Mayo score, MES, steroid use, and advanced therapies. Multivariable logistic regression analysis (Table 3), in which MES was excluded because of the small sample size, revealed that false-negative results in patients with UC were associated positively with age at the time of sample collection and current smokers and negatively with partial Mayo score and advanced therapies. In other words, older patients and current smokers were more likely to have false-negative results, as shown in Figures S2A and B. Patients who received advanced therapies were less likely to have false-negative results (Figure S2C). Notably, in patients with a partial Mayo score of ≥ 5, the false-negative rate was only 2.8%, suggesting the higher sensitivity of V6 Abs for UC diagnosis in patients with more severe symptoms (Figure S2D). Disease extension was not associated with false-negative results of V6 Abs in patients with UC (Table 3 and Figure S2E).

**Table 3:** Multivariable logistic regression analysis of factors associated with false-negative results in patients with UC

	Odds ratio	95% CI	*P* value
Sex (male)	1.033	0.6833–1.561	0.879
**Age at the time of sample collection (per year increase)**	**1.024**	**1.002–1.047**	**0.034**
Smoking status			
Non-smoker	Reference	–	–
Ex-smoker	1.170	0.7130–1.920	0.534
**Current smoker**	**2.550**	**1.422–4.576**	**0.002**
Age at onset (per year increase)	0.9993	0.9774–1.022	0.951
Disease extension			
E3 (pancolitis)	Reference	–	–
E2 (left-sided)	0.7555	0.4566–1.250	0.275
E1 (proctitis)	1.204	0.6640–2.182	0.541
Post-colectomy	1.551	0.3678–6.541	0.550
**Partial Mayo score**	**0.8623**	**0.7495–0.9921**	**0.038**
CRP (per mg/L increase)	1.030	0.6174–1.719	0.909
LRG (per ug/mL increase)	0.9919	0.9425–1.044	0.756
Treatment			
5-aminosalicylic acid or sulfasalazine	0.8471	0.4877–1.472	0.556
Thiopurine	0.9920	0.6258–1.573	0.973
Steroids	0.5787	0.2876–1.165	0.125
**Advanced therapies**	**0.5875**	**0.3765–0.9167**	**0.019**

*UC* ulcerative colitis, *CI* confidence interval, *CRP* C-reactive protein, *LRG* leucine-rich alpha 2 glycoprotein

Bold was made in factors significantly associated with false-negative results

Univariable logistic regression analysis (Table S4) indicated that false-positive results of V6 Abs in patients with CD were associated with age at the time of sample collection, disease location, and CRP. Multivariable logistic regression analysis (Table 4) showed that false-positive results in patients with CD were associated with colonic CD (disease location L2). Indeed, colonic CD had the highest positive rate, followed by ileocolonic and ileal CD (Figure S3A). Clinical activity was not associated with false-positive results of V6 Abs in patients with CD (Table 4 and Figure S3B).

**Table 4:** Multivariable logistic regression analysis of factors associated with false-positive results in patients with CD

	Odds ratio	95% CI	*P* value
Sex (male)	0.9238	0.5493–1.553	0.765
Age at the time of sample collection (per year increase)	0.9864	0.9586–1.015	0.346
Smoking status			
Non-smoker	Reference	–	–
Ex smoker	1.180	0.5817–2.394	0.646
Current smoker	1.578	0.7798–3.194	0.205
Age at onset (per year increase)	0.9895	0.9599–1.020	0.494
Disease location			
L1 (ileal)	Reference	–	–
**L2 (colonic)**	**2.686**	**1.232–5.853**	**0.013**
L3 (ileocolonic)	1.411	0.7779–2.560	0.257
Disease behavior			
B1(non-stricturing, nonpenetrating)	Reference	–	–
B2 (stricturing)	0.8221	0.4610–1.466	0.507
B3 (penetrating)	1.227	0.5910–2.548	0.583
Harvey–Bradshaw index	0.9609	0.8733–1.057	0.414
CRP (per mg/L increase)	1.037	0.7927–1.356	0.793
LRG (per ug/mL increase)	1.028	0.9889–1.069	0.161
Treatment			
5-Aminosalicylic acid or sulfasalazine	1.290	0.7836–2.125	0.317
Thiopurine	0.6798	0.4193–1.102	0.118
Steroids	1.210	0.5092–2.875	0.666
Advanced therapies	1.267	0.7144–2.246	0.419

*CD* Crohn’s disease, *CI* confidence interval, *CRP* C-reactive protein, *LRG* leucine-rich alpha 2 glycoprotein

Bold was made in factors significantly associated with false-positive results

Univariable logistic regression analysis revealed that false-positive results in patients with OGD were not associated with any of the evaluated factors including the serum CRP and leucine-rich alpha 2 glycoprotein levels (Table S5 and Figure S4). We did not perform multivariable analysis due to the small sample size of the patients with OGD.

Lastly, we calculated the value of *I*^2^ (100% × (*Q*−df)/*Q*; where *Q* is Cochran’s heterogeneity statistic, and df is the degrees of freedom) to evaluate the heterogeneity of the diagnostic values among the participating facilities [19]. *I*^2^ values of V6 Ab’s sensitivity for UC and specificities for CD and OGDs were 4.39%, 0.31%, and 0.10%, respectively, indicating little inconsistency [19]. Furthermore, forest plots indicate the consistent UC diagnostic performance of V6 Abs among the centers (Figure S5).

## Discussion

In this study, we evaluated the diagnostic value of V6 Ab for UC using a nationwide cohort. V6 Ab demonstrated a diagnostic sensitivity of 87.7% in 1241 patients with UC and specificities of 82.0% and 87.4% in 796 and 206 patients with CD and OGD, respectively, in 28 Japanese high-volume IBD referral centers.

This study successfully replicated high sensitivity and specificity in the diagnoses of UC by a large-scale multicenter study, which was demonstrated by previous relatively small sample-sized ones [6, 9–13]. Notably, V6 Ab had a high sensitivity of 86.3% for diagnosing UC even in patients with a partial Mayo score of 0, whereas most of the previously reported biomarkers for diagnosis of UC, such as CRP, erythrocyte sedimentation rate, and fecal calprotectin, were evaluated in symptomatic patients [20]. Additionally, patients with moderate to severe UC (a partial Mayo score of ≥ 5) had a much higher sensitivity of 97.2%, which emphasizes the usefulness of V6 Ab for UC diagnosis in clinical settings. Furthermore, this study validated the favorable specificity of V6 Ab for UC diagnosis in patients with CD. Although several biomarkers, including proteinase 3 antineutrophil cytoplasmic antibodies [21] and anti-endothelial protein C receptor antibodies [22], have achieved preliminary results, there are no established diagnostic biomarkers to discriminate UC and CD. The high sensitivity and specificity for UC diagnosis suggest an essential pathophysiological role of this antibody in UC.

Multivariable logistic regression analysis indicated that age at the time of sample collection, current smoker, partial Mayo score, and advanced therapies were associated with false-negative results in the diagnosis of UC among the patients with UC. One previous study indicated a negative correlation between age and V6 Ab titers [11]. The comparison of the disease severity between elderly and non-elderly patients with UC varies among studies. [23, 24] Aging is reported to induce a significant decrease in the number and function of antibody-producing B cells and senescence of B cells [25]. These factors may have affected their V6 Ab titers. Current smokers in this study had a lower positive rate of V6 Ab. Tobacco smoking has been reported to have a protective effect on the development [26] and clinical outcome [27] of UC. Moreover, smoking is associated with decreased IgG production [28]. These reports suggest that smoking might have some impacts on the production of V6 Ab.

In this study, as described above, the positive rate of V6 Abs correlated with the disease activity of UC as in previous reports [6, 10–13], encouraging us to assess V6 Ab as a biomarker for monitoring the activity or predicting the outcome of UC. No previous studies had evaluated the association between V6 Ab and the treatment agent received at the time of sample collection. The present study demonstrated that patients who underwent advanced therapies had a higher positive rate than those that do not. The previous study [11] demonstrated that high V6 Ab levels were associated with a composite of adverse outcomes, including escalation to biologic therapy in recently diagnosed UC. The present study further indicated an association between higher V6 Ab titers and refractoriness of UC.

Although approximately 20% of adult patients with CD were positive for V6 Ab, the clinical characteristics of V6 Ab-positive patients with CD were not assessed in the previous studies [6, 10, 13]. Interestingly, in this study, the positive rate of V6 Ab was higher in colonic CD followed by ileocolonic and ileal CD, and multivariable logistic regression analysis revealed the association between colonic CD and false-positive results. Our previous study on the pediatric population [9] showed that pediatric patients with CD had a higher positive rate of 32.6% compared with adult patients with CD. Antibody-positive patients with CD in the pediatric study were likely to have UC-like endoscopic and histological findings, and 42% had a reviewed diagnosis from UC to CD [9]. A large-scale genotype association study [29] revealed a genetic continuum within IBD and that colonic CD is genetically located between ileal CD and UC. Our present results and previous pediatric data [9] may support their data and may suggest that the high positivity of the antibody in colonic CD is a reflection of the disease characteristics rather than false-positive results.

The specificity of V6 Ab for UC diagnosis in patients with OGD was 87.4% in this study. Most previous studies evaluated the specificity in the healthy control without gastrointestinal disorders and indicated a relatively higher specificity than that of OGDs in this study (96–98%) [10–13]. This may be ascribed to inaccurate diagnosis of OGD and the existence of other diseases in which V6 Ab was applied, such as monogenic IBD [9] and primary sclerosing cholangitis [30], as recently reported. However, univariable logistic regression analysis indicated that no factor was associated with the false-positive results. More large-scale and disease-specific study is necessary to address this issue.

Lastly, we assessed the heterogeneity of the diagnostic value of V6 Ab for UC among the participating centers. Both *I*^2^ values and forest plots of V6 Ab sensitivity for UC and specificities for CD and OGDs indicated the consistency of V6 Abs as a biomarker for UC diagnosis.

This study has limitations. Data on fecal biomarkers in IBD and endoscopic activity in CD are lacking. Another limitation may be that participating centers were limited to Japan. However, since several studies in Western countries replicated the usefulness of V6 Ab for the diagnosis of UC [10–13], our study with a Japanese nationwide cohort appears valuable for comparison.

In conclusion, the diagnostic value of V6 Ab for UC was validated in the Japanese large-scale nationwide multicenter study. Further large-scale investigations among people of other ethnicities are necessary.

## Supplementary Information

Below is the link to the electronic supplementary material.

Supplementary file1 (PDF 1128 KB)

## Abbreviations

CD: Crohn’s disease
CRP: C-reactive protein
ELISA: Enzyme-linked immunosorbent assay
IBD: Inflammatory bowel disease
IBDU: Inflammatory bowel disease unclassified
MES: Mayo endoscopic subscore
OGD: Other gastrointestinal disorder
SD: Standard deviations
UC: Ulcerative colitis
V6 Ab: Anti-integrin αvβ6 antibody
