# Publisher Correction: Anti-integrin αvβ6 antibody as a biomarker for diagnosing ulcerative colitis: a nationwide multicenter validation study

**DOI:** 10.1007/s00535-024-02203-x

**Published:** 2024-12-27

**Authors:** Makoto Okabe, Shuji Yamamoto, Masahiro Shiokawa, Tadakazu Hisamatsu, Hajime Yamazaki, Risa Nakanishi, Kensuke Hamada, Hiroki Kitamoto, Takeshi Kuwada, Norimitsu Uza, Aki Sakatani, Toshimitsu Fujii, Masashi Ohno, Minoru Matsuura, Tomoyoshi Shibuya, Naoki Ohmiya, Makoto Ooi, Namiko Hoshi, Kei Moriya, Kiichiro Tsuchiya, Yoshiharu Yamaguchi, Reiko Kunisaki, Masahiro Takahara, Tomohisa Takagi, Tetsuo Takehara, Fumihito Hirai, Kazuki Kakimoto, Motohiro Esaki, Hiroshi Nakase, Fukunori Kinjo, Takehiro Torisu, Shuji Kanmura, Kazuyuki Narimatsu, Katsuyoshi Matsuoka, Hiroto Hiraga, Kaoru Yokoyama, Yusuke Honzawa, Makoto Naganuma, Masayuki Saruta, Yuzo Kodama, Tsutomu Chiba, Hiroshi Seno

**Affiliations:** 1 Department of Gastroenterology and Hepatology, Graduate School of Medicine, Kyoto University, 54 Shogoin Kawahara-cho, Sakyo-ku, Kyoto 606-8507 Japan; 2 Department of Gastroenterology and Hepatology, Kyorin University School of Medicine, Mitaka-shi, Tokyo 181-8611 Japan; 3 Section of Clinical Epidemiology, Department of Community Medicine, Graduate School of Medicine, Kyoto University, Kyoto, 606-8507 Japan; 4 Division of Gastroenterology, Department of Internal Medicine, Asahikawa Medical University, Asahikawa, 078-8510 Japan; 5 Department of Gastroenterology and Hepatology, Tokyo Medical and Dental University, Tokyo, 113-8519 Japan; 6 Department of Medicine, Shiga University of Medical Science, Otsu, 520-2192 Japan; 7 Department of Gastroenterology, Juntendo University School of Medicine, Tokyo, 113-8421 Japan; 8 Department of Advanced Endoscopy, Fujita Health University School of Medicine, Toyoake, 470-1192 Japan; 9 Division of Gastroenterology, Department of Internal Medicine, Graduate School of Medicine, Kobe University, Kobe, 650-0017 Japan; 10 Department of Gastroenterology, Nara Prefecture General Medical Center, Nara, 630-8581 Japan; 11 Department of Gastroenterology, Institute of Medicine, University of Tsukuba, Tsukuba, 305-8576 Japan; 12 Division of Gastroenterology, Department of Internal Medicine, Aichi Medical University School of Medicine, Nagakute, 480-1195 Japan; 13 Inflammatory Bowel Disease Center, Yokohama City University Medical Center, Yokohama, 232-0024 Japan; 14 Department of Gastroenterology and Hepatology, Dentistry and Pharmaceutical Sciences, Okayama University Graduate School of Medicine, Okayama, 700-8558 Japan; 15 Department of Molecular Gastroenterology and Hepatology, Kyoto Prefectural University of Medicine, Kyoto, 602-8566 Japan; 16 Department of Gastroenterology and Hepatology, Osaka University Graduate School of Medicine, Suita, Osaka 565-0871 Japan; 17 Department of Gastroenterology, Faculty of Medicine, Fukuoka University, Fukuoka, 814-0180 Japan; 18 Second Department of Internal Medicine, Osaka Medical and Pharmaceutical University, Takatsuki, 569-8686 Japan; 19 Division of Gastroenterology, Department of Internal Medicine, Faculty of Medicine, Saga University, Saga, 849-8501 Japan; 20 Department of Gastroenterology and Hepatology, Sapporo Medical University School of Medicine, Sapporo, 060-8543 Japan; 21 Center for Gastroenterology, Urasoe General Hospital, Urasoe, 901-2102 Japan; 22 Department of Medicine and Clinical Science, Graduate School of Medical Sciences, Kyushu University, Fukuoka, 812-8582 Japan; 23 Division of Digestive and Lifestyle Diseases, Kagoshima University Graduate School of Medical and Dental Sciences, Kagoshima, 890-8520 Japan; 24 Department of Internal Medicine, National Defence Medical College, Tokorozawa, 359-8513 Japan; 25 Division of Gastroenterology and Hepatology, Department of Internal Medicine, Toho University Sakura Medical Center, Sakura, 285-8741 Japan; 26 Department of Gastroenterology and Hematology, Hirosaki University Graduate School of Medicine, Hirosaki, 036-8563 Japan; 27 Department of Gastroenterology, Kitasato University School of Medicine, Kanagawa, 252-0375 Japan; 28 Division of Gastroenterology and Hepatology, Third Department of Internal Medicine, Kansai Medical University, Osaka, 573-1010 Japan; 29 Division of Internal Medicine, Department of Gastroenterology and Hepatology, The Jikei University School of Medicine, Tokyo, 105-8471 Japan; 30 Department of Gastroenterology and Hepatology, Kansai Electric Power Hospital, Fukushima-ku, Osaka, 553-0003 Japan

**Publisher Correction: J Gastroenterol** 10.1007/s00535-024-02176-x

In the original publication of the article, Tadakazu Hisamatsu was incorrectly associated with the "Department of Gastroenterology and Hepatology, Graduate School of Medicine, Kyoto University, 54 Shogoin Kawahara-cho, Sakyo-ku, Kyoto, 606-8507, Japan." The publisher extends a sincere apology for any inconvenience this error may have caused.

The original article has been corrected.

