# Supplementary material for: Anti-integrin αvβ6 antibody as a biomarker for diagnosing ulcerative colitis: a nationwide multicenter validation study
Source: J Gastroenterol. 2024 Nov 28;60(1):86–95. doi: 10.1007/s00535-024-02176-x (PMC11717824; doi:10.1007/s00535-024-02176-x)
Supplement: Supplementary file 1 — Supplementary file1 (PDF 1128 KB) [file 535_2024_2176_MOESM1_ESM.pdf]

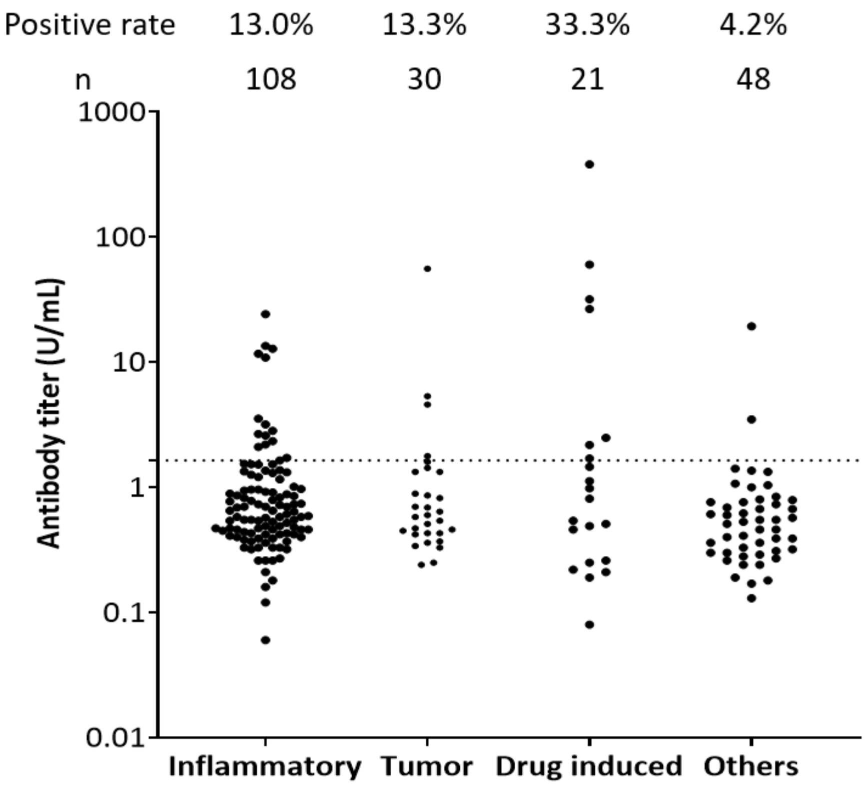

Figure S1. Comparison of anti-integrin αvβ6 antibody titers among patients with other gastrointestinal diseases.

The cut-off value of 1.64 U/mL is indicated by a dashed line. n: Total number of patients in each group.

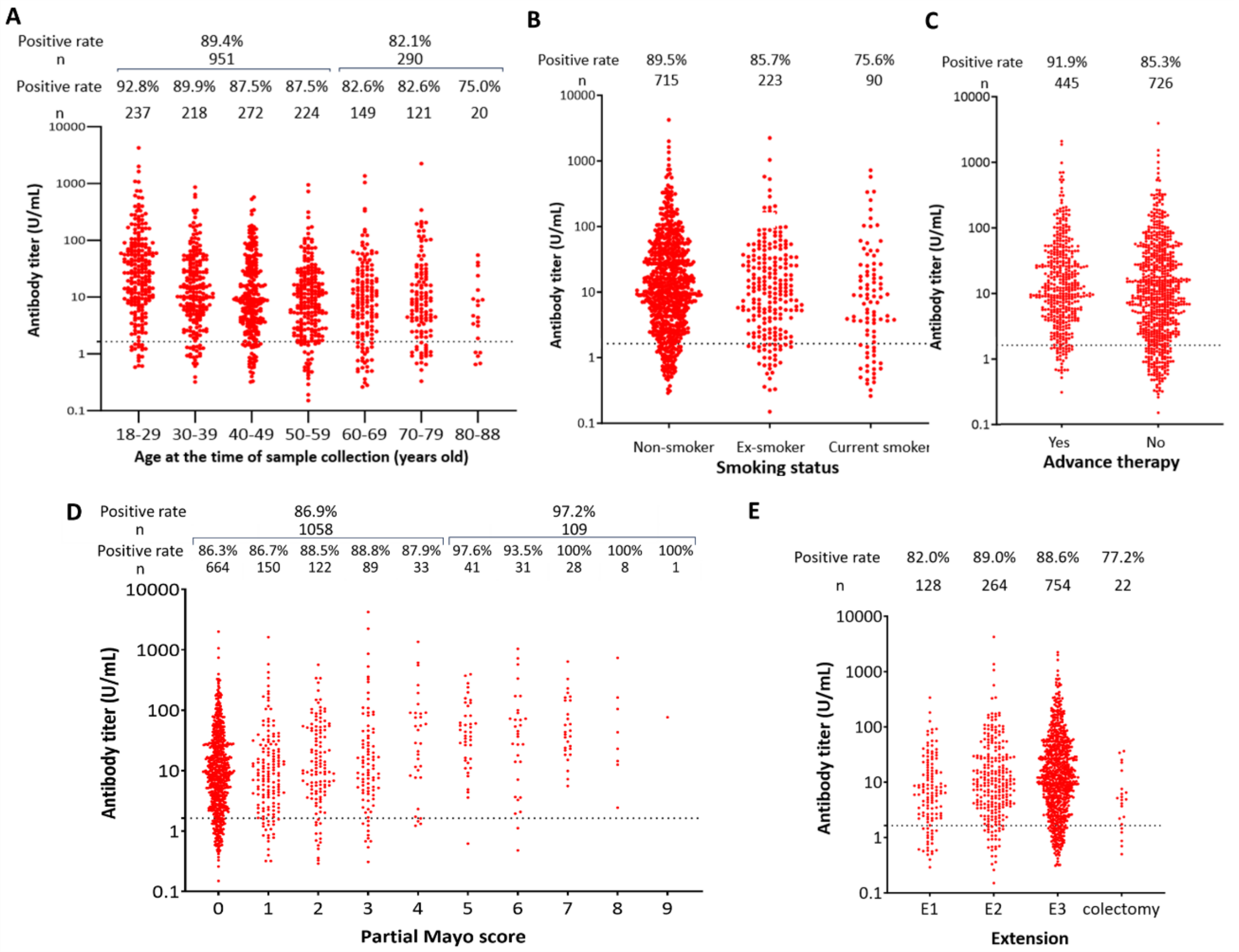

Figure S2. Comparison of anti-integrin αvβ6 antibody titers among patients with ulcerative colitis with different ages at the time of sample collection (A), smoking status (B), treatment modalities (C), clinical disease activities (D), and disease extension (E). The chi-squared test indicated statistical differences in age (less than 60-year-old *vs* 60-year-old or more, A; $p<0.001$), smoking status (B; $p<0.001$), treatment (C; $p<0.001$), and clinical activity (partial Mayo score of 0-4 *vs* 5-9, D; $p<0.01$), but not in disease extension (E; $p=0.071$).

The cut-off value of 1.64 U/mL is indicated by a dashed line. n: Total number of patients in each group.

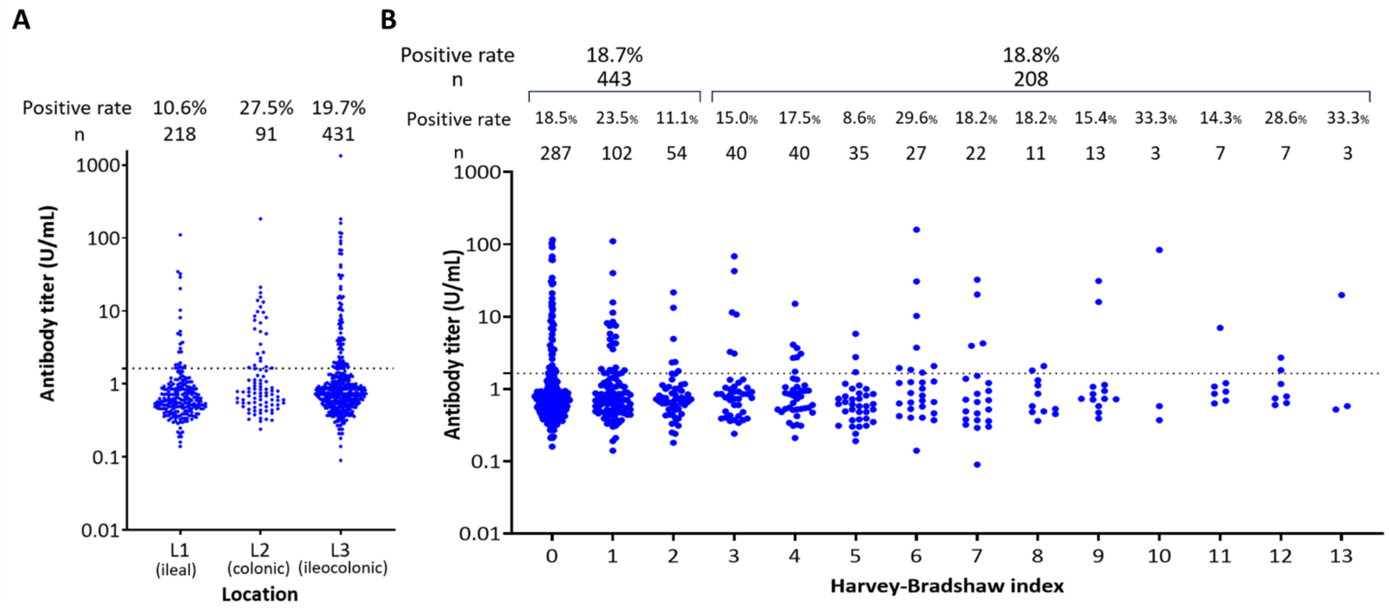

Figure S3. Comparison of anti-αvβ6 integrin antibody titers among the different types of Crohn's disease based on their location (A) and clinical activities (B)

(A) Serum antibody titers in patients with ileal (L1), colonic (L2), and ileocolonic (L3) Crohn's disease were compared. The positive rate in the patients with ileal or ileocolonic Crohn's disease was 16.6% (i.e., the specificity was 83.4%). (B) Serum antibody titers were compared between the patients with clinical remission (Harvey-Bradshaw index of 3 or less) and those with active disease (Harvey-Bradshaw index of 4 or more).

The cut-off value of 1.64 U/mL is indicated by a dashed line. n: Total number of patients in each group.

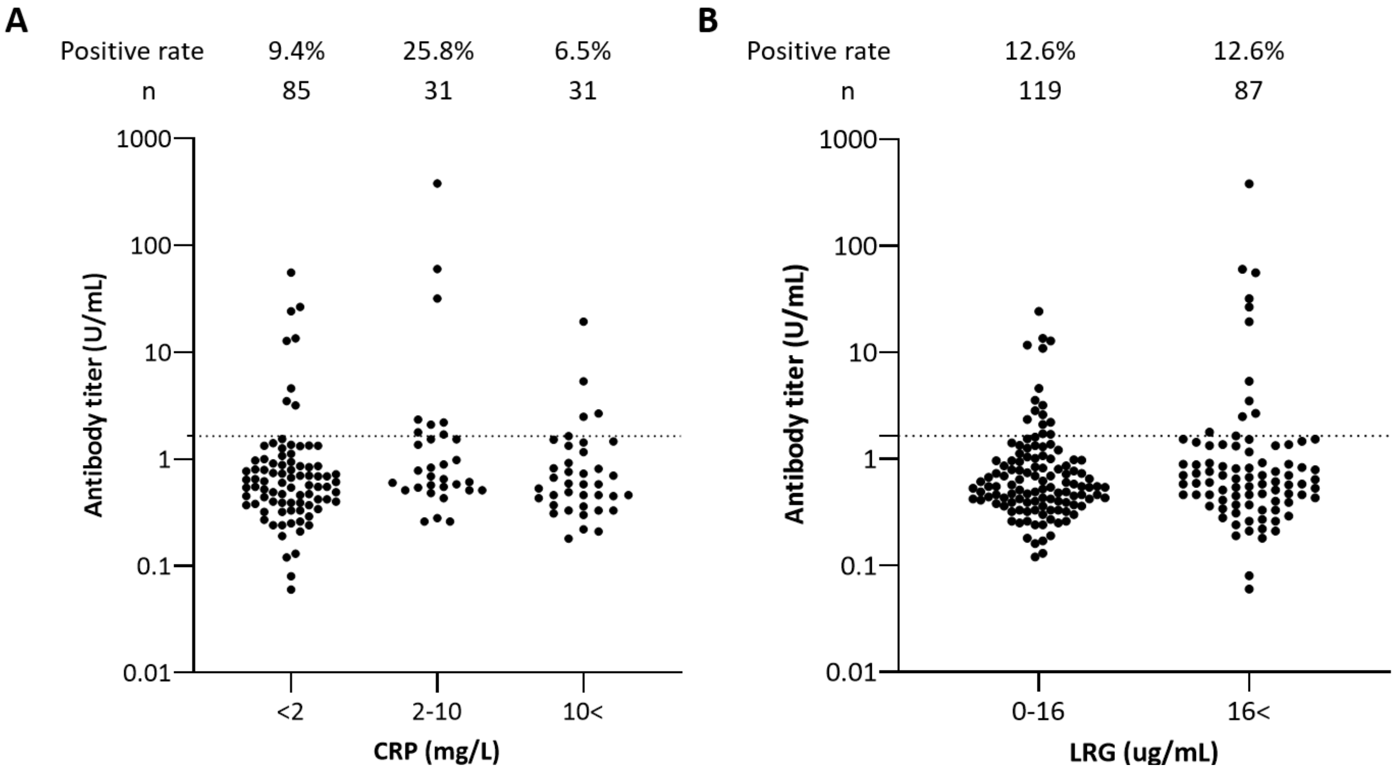

Figure S4. Comparison of anti-integrin αvβ6 antibody titers among patients with other gastrointestinal diseases with different inflammatory activities based on serum CRP (A) and LRG (B) levels.

The cut-off value of 1.64 U/mL is indicated by a dashed line. n: Total number of patients in each group, CRP: C-reactive protein, LRG: leucine-rich alpha 2 glycoprotein

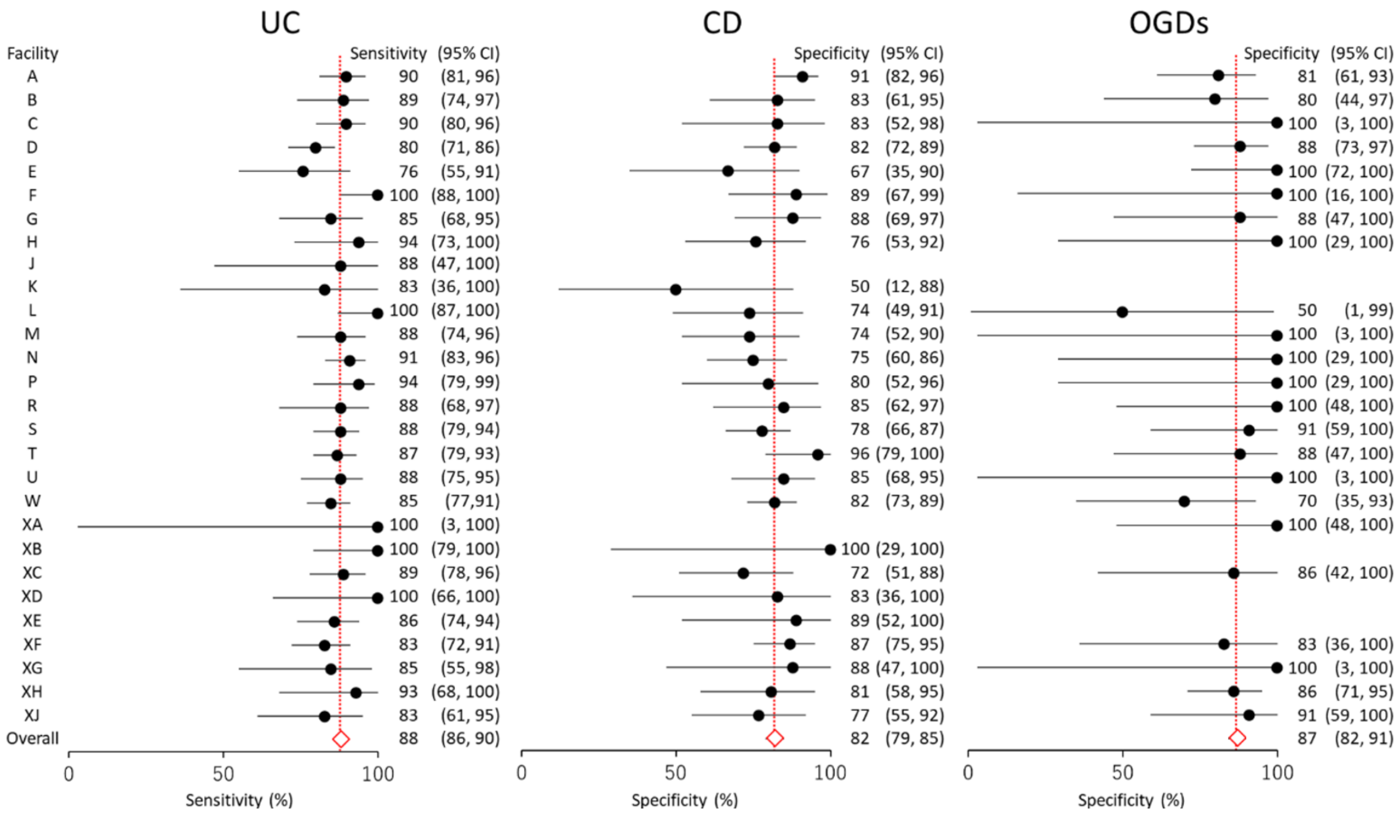

Figure S5. Forest plots of the diagnostic value of anti-αvβ6 integrin antibody for UC in each participating facility

UC: ulcerative colitis, CD: Crohn's disease, OGD: other gastrointestinal diseases.

Table S1. Participating facilities

Aichi Medical University Hospital	Nara Prefecture General Medical Center
Asahikawa Medical University Hospital	National Defence Medical College Hospital
Fujita Health University Hospital	Okayama University Hospital
Fukuoka University Hospital	Osaka Medical and Pharmaceutical University Hospital
Hirosaki University Hospital	Osaka University Hospital
Jikei University Hospital	Saga University Hospital
Juntendo University Hospital	Sapporo Medical University Hospital
Kagoshima University Hospital	Shiga University of Medical Science Hospital
Kansai Medical University Hospital	Toho University Sakura Medical Center
Kitasato University Hospital	Tokyo Medical and Dental University Hospital
Kobe University Hospital	University Hospital, Kyoto Prefectural University of Medicine
Kyorin University Hospital	University of Tsukuba Hospital
Kyoto University Hospital	Urasoe General Hospital
Kyushu University Hospital	Yokohama City University Medical Center

Table S2. Diagnostic criteria for each of the other gastrointestinal disorders

Behcet's disease	Diagnosed based on the criteria for diagnosing Behçet's disease.[1]
Chronic enteropathy associated with *SLCO2A1* gene (CEAS)	Diagnosed based on the Japanese criteria for diagnosing chronic enteropathy associated with the *SLCO2A1* gene.[2]
Cronkhite-Canada syndrome	Diagnosed based on the Japanese criteria for diagnosing Cronkhite–Canada syndrome.[3]
Familial Mediterranean fever	Diagnosed based on the Japanese criteria for diagnosing familial Mediterranean fever.[4]
IgA vasculitis	Diagnosed based on the criteria for IgA vasculitis[5)]
Takayasu's arteritis	Diagnosed based on the criteria for diagnosing Takayasu's arteritis.[5]
Eosinophilic granulomatosis with polyangiitis	Diagnosed based on the criteria for diagnosing Eosinophilic granulomatosis with polyangiitis.[6]
Eosinophilic gastroenteropathy	Diagnosed based on the presence of eosinophilic infiltration of the gastrointestinal tract on biopsy and/or eosinophilic ascitic fluid, lack of involvement of other organs, and absence of other causes of intestinal eosinophilia
Congenital immunodeficiency	Diagnosed based on immune function tests and/or genetic examinations
Colonic adenocarcinomas or adenomas	Diagnosed based on histological findings.
Neuroendocrine tumors	Diagnosed based on histological findings.
Gastrointestinal lymphoma	Diagnosed based on histological findings.
Immune-related adverse events (irAE)	Diagnosed based on the presence of diarrhea, the use of immune checkpoint inhibitor, and the absence of other causes of intestinal disorders
Olmesartan associated enteropathy	Diagnosed based on the criteria for diagnosing Olmesartan-associated enteropathy.[7]
Non-steroidal anti-inflammatory drug-induced enteropathy	Diagnosed based on endoscopic findings in patients using non-steroidal anti-inflammatory drugs.
Microscopic colitis	Diagnosed based on histological findings in patients with watery diarrhea
Infectious enterocolitis	Diagnosed based on the presence of fever, diarrhea of rapid onset, and bloody stool, and the identification of etiologic bacteria
Radiation enterocolitis	Diagnosed based on endoscopic findings in patients with a history of radiation therapy
Ischemic colitis	Diagnosed based on colonoscopy or radiographic imaging in patients with abdominal pain and bloody stool
Irritable bowel syndrome	Diagnosed based on the Rome IV criteria.[8]

Colonic diverticular disease	Diagnosed based on colonoscopy or radiographic imaging.
Gastroesophageal reflux disease	Diagnosed based on endoscopic findings.
Non-specific enterocolitis	Diagnosed based on endoscopic findings.
Malabsorption syndrome	Diagnosed based on symptoms and laboratory data.
Amyloidosis	Diagnosed based on histologic findings.
Phlebosclerosis	Diagnosed based on endoscopic, radiographic, and histologic findings.[9]
Bowel obstruction	Diagnosed based on radiographic imaging.

1) Wechsler B, Davatchi F, Mizushima Y, et al. Criteria for diagnosis of Behçet's disease. International Study Group for Behçet's Disease. Lancet 1990;335:1078-80.
2) Umeno J, Esaki M, Hirano A, et al. Clinical features of chronic enteropathy associated with SLCO2A1 gene: a new entity clinically distinct from Crohn's disease. J Gastroenterol 2018;53: 907-15.
3) Watanabe C, Komoto S, Tomita K, et al. Endoscopic and clinical evaluation of treatment and prognosis of Cronkhite-Canada syndrome: a Japanese nationwide survey. J Gastroenterol 2016;51:327-36.
4) Papadopoulos VP, Giaglis S, Mitroulis I, Ritis K. The population genetics of familial Mediterranean fever: a meta-analysis study. Ann Hum Genet 2008;72:752-61.
5) Jennette JC, Falk RJ, Bacon PA, et al. 2012 revised international chapel hill consensus conference nomenclature of vasculitides. Arthritis Rheum 2013;65:1-11.
6) Masi AT, Hunder GG, Lie JT, et al. The American College of Rheumatology 1990 criteria for the classification of Churg-Strauss syndrome (allergic granulomatosis and angiitis). Arthritis Rheum 1990;33:1094-100.
7) Rubio-Tapia A, Herman ML, Ludvigsson JF, et al. Severe spruelike enteropathy associated with olmesartan. Mayo Clin Proc 2012;87:732-8.
8) Lacy BE, Mearin F, Chang L, et al. Bowel disorders. Gastroenterology 2016;150:1393-1407.
9) Iwashita A, Yao T, Schlemper RJ et al. Mesenteric phlebosclerosis: a new disease entity causing ischemic colitis. Dis Colon Rectum 2003;46:209-20.

Table S3. Univariate logistic regression analysis of factors associated with false-negative results in patients with ulcerative colitis

	Odds ratio	95% CI	*P* value
**Sex** (Male)	1.129	0.8026 – 1.589	0.485
**Age at the time of sample collection (per year increase)**	**1.021**	**1.011 – 1.032**	**<0.001**
**Smoking status**			
Non-smoker	Reference	-	-
Ex-smoker	1.4297	0.9168 – 2.2294	0.115
Current smoker	**2.761**	**1.614 – 4.723**	**<0.001**
**Age at onset (per year increase)**	**1.016**	**1.006 – 1.027**	**0.003**
**Disease extension**			
E3 (pancolitis)	Reference	-	-
E2 (left-sided)	0.9585	0.6134 – 0.8221	0.852
E1 (proctitis)	**1.701**	**1.0278 – 2.817**	**0.039**
post colectomy	2.285	0.7363 – 5.943	0.113
**Partial Mayo score**	**0.8330**	**0.7400 – 0.9378**	**0.003**
**Mayo endoscopic subscore**	**0.4281**	**0.2472 – 0.6709**	**<0.001**
**CRP (per one-mg/L increase)**	0.8068	0.5478 – 1.188	0.277
**LRG (per one-ug/mL increase)**	0.9871	0.9584 – 1.017	0.387
**Treatment**			
5-aminosalicylic acid or sulfasalazine	0.9286	0.5851 – 1.474	0.753
Thiopurine	0.8953	0.5920 – 1.354	0.601
Steroids	**0.4171**	**0.2146 – 0.8109**	**0.010**
Advanced therapies	**0.5092**	**0.3421 – 0.7579**	**0.001**
**Duration of serum storage (per month increase)**	1.002	0.9888 – 1.019	0.793

CI: confidence interval, CRP: C-reactive protein, LRG: leucine-rich alpha 2 glycoprotein

Table S4. Univariate logistic regression analysis on factors associated with false-positive results in patients with Crohn's disease

	Odds ratio	95% CI	*P* value
**Sex** (Male)	0.8654	0.5900 – 1.269	0.459
**Age at the time of sample collection (per year increase)**	**0.9861**	**0.9729 – 0.9994**	**0.041**
**Smoking status**			
Non-smoker	Reference	-	-
Ex-smoker	0.7419	0.3935 – 1.399	0.356
Current smoker	1.151	0.6218 – 2.130	0.655
**Age at onset (per year increase)**	0.992450	0.9780 – 1.007	0.312
**Disease location**			
L1 (ileal)	Reference	-	-
L2 (colonic)	**3.212**	**1.708 – 6.038**	**<0.001**
L3 (ileocolonic)	**2.083**	**1.272 – 3.410**	**0.004**
**Disease behavior**			
B1 (non-stricturing, nonpenetrating)	Reference	-	-
B2 (stricturing)	0.7460	0.4952 – 1.124	0.161
B3 (penetrating)	0.8305	0.4627 – 1.491	0.534
**Harvey-Bradshaw index**	1.001	0.9372 – 1.070	0.971
**CRP (per mg/L increase)**	**1.214**	**1.025 – 1.439**	**0.025**
**LRG (per ug/mL increase)**	1.022	0.9992 – 1.045	0.058
**Treatment**			
5-aminosalicylic acid or sulfasalazine	0.9646	0.6436 – 1.446	0.862
Thiopurine	0.8287	0.5585 – 1.230	0.351
Steroids	1.214	0.5885 – 2.505	0.600
Advanced therapies	1.435	0.8868 – 2.323	0.141
**Duration of serum storage (per month increase)**	0.9912	0.9765 – 1.008	0.269

CI: confidence interval, CRP: C-reactive protein, LRG: leucine-rich alpha 2 glycoprotein

Table S5. Univariate logistic regression analysis on factors associated with false-positive results in patients with other gastrointestinal diseases

	Odds ratio	95% CI	*P* value
**Sex** (Male)	0.7333	0.3194 – 1.684	0.465
**Age at the time of sample collection (per year increase)**	0.9806	0.9580 – 1.004	0.100
**Smoking status**			
Non-smoker	Reference	-	-
Ex-smoker	0.6250	0.1638 – 2.384	0.491
Current smoker	0.4167	0.0501 – 3.468	0.418
**Age at onset (per year increase)**	0.9902	0.9692 – 1.012	0.365
**Disease**			
Inflammatory diseases	Reference	-	-
Tumors	1.033	0.3133 – 3.406	0.958
Drug-induced gastrointestinal disorders	2.878	0.9493 – 8.723	0.062
Others	0.2919	0.0637 – 1.339	0.113
**CRP (per mg/L increase)**	1.048	0.9068 – 1.211	0.527
**LRG (per ug/mL increase)**	1.007	0.9654 – 1.050	0.749
**Duration of serum storage (per month increase)**	1.019	0.9744 – 1.132	0.572

CI: confidence interval, CRP: C-reactive protein, LRG: leucine-rich alpha 2 glycoprotein
